# Latina mothers’ perspectives on adverse experiences and protection of Latinx youth in an agricultural community

**DOI:** 10.1186/s12889-023-14993-5

**Published:** 2023-02-02

**Authors:** Deepika D. Parmar, Alexandra M. Minnis, Elodia Caballero, Melissa Zerofsky, Megan Comfort, Marissa Raymond-Flesch

**Affiliations:** 1 Division of Adolescent and Young Adult Medicine, Department of Pediatrics, University of California, San Francisco, San Francisco, CA USA; 2 RTI International, Berkeley, CA USA; 3 School of Medicine, University of California, San Francisco, San Francisco, CA USA; 4 Department of Pediatrics, University of California, San Francisco, San Francisco, CA USA

**Keywords:** Latinx, Immigrant, Adverse Childhood Experiences, Trauma, Resilience

## Abstract

**Background:**

Adverse Childhood Experiences (ACEs) are a measure of childhood toxic stress that have a dose-dependent relationship with many adult health outcomes. While ACEs have been validated across diverse populations to measure neglect, abuse, and family dysfunction, they do not specifically assess trauma related to racism/xenophobia and immigration. 54% of Latinx youth in the United States are immigrants or children of immigrants and a large group with potentially unmeasured trauma. This study looks beyond ACEs to identify adverse and protective factors for healthy development among Latinx youth in an agricultural community through the perspectives of their mothers.

**Methods:**

Twenty mothers of adolescent participants in *A Crecer*: the Salinas Teen Health Study (a prospective cohort study of 599 adolescents) completed semi-structured interviews in Spanish. Interviews focused on mothers’ perspectives on community resources, parenting strategies, parenting support systems, and their future aspirations for their children. Four coders completed iterative rounds of thematic coding drawing from published ACEs frameworks (original ACEs, community ACEs) and immigrant specific adverse events arising from the data.

**Results:**

Mothers in this study reported adverse experiences captured within community-level ACEs but also distinct experiences related to intergenerational trauma and immigrant-related adversities. The most cited community-level ACEs were housing instability and community violence. Immigrant related adversities included experiences of systemic racism with loss of resources, political instability limiting structural resources, and language-limited accessibility. These were exacerbated by the loss of family supports due to immigration related family-child separation including deportations and staggered parent–child migration. Having experienced intergenerational trauma and systemic oppression, mothers discussed their strategies for building family unity, instilling resilience in their children, and improving socioeconomic opportunities for their family.

**Conclusions:**

Latina mothers shared the impacts of immigrant-related experiences on systemic inequities in the United States which are currently missing from the ACEs framework. Immigrant specific adverse events include language-limited accessibility, or family-child separations, and policies impacting structural resources for immigrant families. Mothers highlighted their capacity to build resilience in their children and buffer impacts of systemic racism. Community-tailored interventions can build on this foundation to reduce health disparities and promote health equity in this population.

## Background

The original Adverse Childhood Experiences (ACEs) framework identified key aspects of trauma that increase the risk of 7 of the 10 leading causes of death in the United States and shorten life expectancy in a dose dependent manner [1–5]. This important framework, originally studied across categories of abuse, neglect, and family dysfunction, was further broadened to include the impact of community-level adversities (i.e. community violence, bullying, discrimination) in socioeconomically and racially diverse populations [6, 7]. These community-level Expanded ACEs, are pervasive across sociodemographic groups, but more prevalent in racial and sexual minority populations where they may exacerbate health inequities across generations [6]. The Expanded ACEs were extended to include food and housing insecurity and are now used for screening in primary care clinics [8, 9]. The original ACEs are a screening tool that focuses on risk factors and does not capture how resilience may influence outcomes. Subsequent studies indicate that it is possible to buffer against the damaging effects of ACEs with interventions that build resilience in youth [10–12]. In addition, the Expanded ACEs identify structural domains of adversity such as food and housing insecurity that are intervenable beyond individual resilience-building. While the original and the Expanded ACEs, identify many adverse events, they do not encompass many of the developmental and public policy stressors that minorities, immigrants, and children in immigrant communities experience in the United States (US) [13].

One quarter of children in the US are born to immigrant parents, including 54% of Latinx children [14]. Yet, few studies review ACEs in this population, and the results show differential impacts. Some studies indicate that despite typically having less education, lower socioeconomic status, and potential language barriers, first and second generation immigrant families experience fewer original ACEs compared with native-born American adults in the US [15, 16]. Among Latinx children, those in immigrant families had significantly lower odds of ACEs compared to children born to US citizen parents, in a nationally represented study [17]. Other data suggest that this difference is not reflective of a true health advantage alone, but rather due to variability in cultural perception and reporting of adversity between immigrant and US-native families [16]. The ACE-I study, which focused on first-generation child immigrants, was on the vanguard of describing immigrant-related ACEs; however, it excludes U.S. born children. Of the estimated 17.9 million Latinx youth under 18 in the U.S., 52% have a parent born outside of the U.S. and about one quarter have a parent without documentation; all of these children are also likely to be impacted by immigration policies [18–20].

Nonetheless, numerous studies have documented the mental and physical health impacts of immigration policies which create an environment of political precarity and continuous threat of family separation, deportation, and discrimination [21–23]. Immigrant youth from families vulnerable to deportation compared to youth with low to moderate vulnerability, had higher anxiety levels, sleep problems and blood pressure changes associated with U.S. immigration policy changes in 2016 [21]. Moreover, families with undocumented parents and U.S.-born children report poorer child global health rating compared to U.S.-born Latinx families and mixed-status legal permanent resident families, especially when they perceived their state of residence as having punitive immigrant policies [24].

Neither the original ACEs nor the expanded ACEs capture these adverse experiences. Despite growing adversities, immigrant families have continued to improve their life-course trajectories through resilience. Resilience is built by the combination of supportive relationships, adaptive skill-building, and positive experiences influenced by families and communities [25–27]. A safe, stable, and nurturing relationship with a supportive adult can be the strongest component to building resilience [25]. One example of resilience-building found in Latinx families is the concept of Familismo [28]. Familismo’s features including pride, belonging, and obligation to family [28]. Familismo persists in Latinx communities regardless of length of time in the US [28]. It is associated with increased resilience and reduced internalizing mental health symptoms [28–30]. The American Academy of Pediatrics affirms the critical role of family and community relationships on child health, calling for integration of attention to these family and community relationships both vertically (by including primary, secondary, and tertiary preventions) and horizontally (by including public service sectors beyond health care) [27].

To better understand the role of ACEs and potentially capture missing measures in families, communities and intergenerational experiences, research supports shifting the focus from the individual child to a broader appreciation of the family support systems [31–33]. Neuroscience research is now showing that trauma in one generation can be transmitted through epigenetic mechanisms to their offspring highlighting the importance of family and social context for childhood development [34]. Through research engagement with parents, we can gather extended information on a child’s history with richer context and intergenerational impacts. In fact, studies have shown that even mothers’ ACEs matter—a history of trauma in mothers, shows measurable impacts with association of early initiation of smoking in their children [35]. However, mothers who experience violence often buffer against the negative impacts on their children by acting as ‘emotional anchors’, demonstrating adaptive coping mechanisms that lead them to act warmly and responsively towards their children and mediate their children’s distress [36].

In this study, we used qualitative interviews with mothers to explore the influences of family structure, intergenerational trauma and resilience, and community-level resources on early adolescents in an immigrant agricultural community. Our aim was to identify immigrant-relevant gaps in the original and Expanded ACEs frameworks by examining both adverse experiences and protective experiences for these Latinx youth. Through interviews with mothers, we identified how they build resilience in their adolescents through parenting strategies, accessing community resources, immigrating with children, and navigating systemic barriers for immigrants.

## Methods

The setting and study design of the *A Crecer*study has been described elsewhere [26, 37]. In brief, it is a community-engaged, mixed methods, longitudinal cohort study that followed a cohort of 599 primarily Latinx adolescents during their transition from eighth grade into high school. Eighty-five percent of the A Crecer cohort were US born. The study takes place in the agricultural community of Salinas, CA where 31% of residents are under 18, 79% are Latinx (primarily Mexican-origin) and 37% are first-generation immigrants [38].

### Study design and population

The analysis presented here focuses on twenty semi-structured interviews of mothers of youth in the *A Crecer* cohort. These interviews were conducted in 2019 in-person in a private room at the study office in downtown Salinas. Each was about 60 min in duration.

Inclusion Criteria: Mothers of participants were purposefully sampled to include a balance of participants based on: self-identified gender of their adolescent, educational attainment (completion of high school or less than high school), and youth’s immigrant generation. This information was available because the Mother’s children were original participants in the parent study, A Crecer and we were able to select them for recruitment based on the demographic information that their children provided. All interviews with mothers were conducted by bilingual and bicultural members of the research team in Spanish or English, as per participant preference.

These interviews focused on mothers’ perspectives on community resources and strengths in Salinas, parenting strategies, parenting support systems, strengths of their children and their future aspirations for their children. Mothers received $20 for their time. The RTI and UCSF Institutional Review Boards approved all study activities and all methods and protocols were carried out in accordance with their relevant guidelines and regulations. Mothers provided written informed consent in the language of their choice.

#### Analysis

Interviews were translated and transcribed verbatim. A team of four researchers coded the interview transcripts with two additional senior investigators (AM and MC) reviewing the codebook and offering insights on emerging themes. Coders completed iterative rounds of thematic coding. Codes were initially developed a priori based on the PEARLS Study ACEs framework [8, 9]. However, in addition to creating codes based on ACEs, the study team also engaged in open coding allowing in-vivo codes to arise based on grounded theory approach developed by Glaser and Straus to allow expansion of codes and subcodes unique to immigrant experiences [39]. For these in vivo codes, initial rounds of thematic analysis informed memos which were discussed and distilled into codes at team meetings. Two transcripts were then coded by all team members resulting in further refinement of the code book and assessment of inter-coder agreement; remaining transcripts were each coded by two team members. The coding team was made up of 1 Latina-identifying 1^st^ generation immigrant woman, 1 Latina-identifying 2^nd^ generation immigrant woman, 1 South-Asian identifying 1^st^ generation immigrant woman and 1 white-identifying women. The team spent weekly and biweekly meetings discussing biases, reflecting on our identities as we engaged in coding. We then took our findings back to the community members and broader research team to ensure fidelity and interpretation was consistent with community views.

Additionally, as we described, we attended to coding fidelity as follows, “Two transcripts were then coded by all team members resulting in further refinement of the code book and assessment of inter-coder agreement; remaining transcripts were each coded by two team members.”

## Results

The characteristics of the study sample (*n *= 20) are shown in Table 1. Mothers’ mean age was 41.8 years and ranged from ages 32 to 53. All mothers in the sample were primarily Spanish-speaking and therefore all of the interviews were conducted in Spanish per their preference. Eighty percent had less than a high school education. All were immigrants who came to the United States an average of 19.8 years prior to the study.

**Table 1:** Sociodemographic characteristics of mothers of A Crecer study participants *n* = 20

Demographics	Mean (range)	n(%)
Age	42 (32–53)	
Years lived in US	21 (14–34)	
Age at Migration	20 (13–29)	
Mother's education		
less than high school		16 (80%)
high school only		1 (5%)
post high school		3 (15%)
Occupation		
agriculture		15 (75%)
not working		3 (15%)
working other		2 (10%)
Dependents in the home	3 (1–5)	

**Fig. 1:**
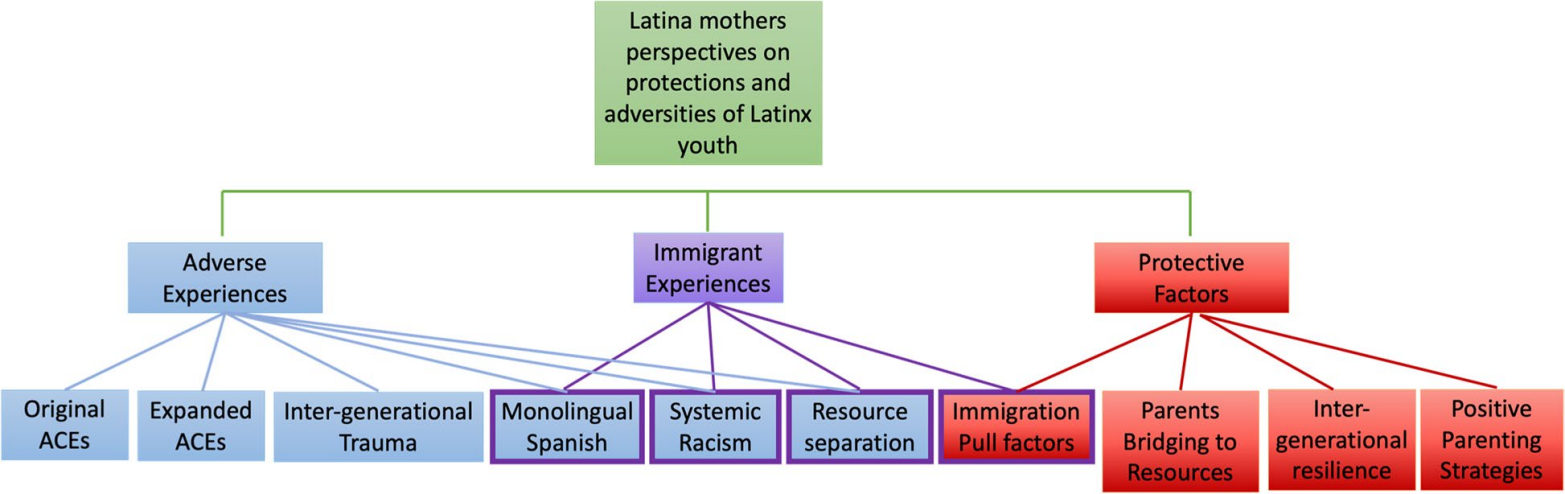
Coding tree representing the codes, categories and themes that emerged in the focus groups. Immigration experiences codes frequency overlapped with adverse experiences and protective factors as indicated by the purple outlines on some of the codes

The team identified key themes (Fig. 1) including Traumatic Experiences, Protective Factors, and Immigration. Traumatic Experiences were subdivided into Original Adverse Childhood Experiences (Table 2), Expanded ACEs (Table 2), and Intergenerational Trauma. The team subdivided Protective Factors into Intergenerational Resilience, Types of Parental Support, Positive Parenting Strategies, Parents Bridging to Resources, and Money. Immigration overlapped both protective and adverse experiences and therefore these themes were subdivided further.

**Table 2:** Adverse childhood experiences categories and examples from Latina mothers

ESTABLISHED ADVERSE CHILDHOOD EXPERIENCES	EXAMPLES
Original Adverse Childhood Experiences: Harm	
PHYSICAL: Child experienced or was threatened by physical abuse (pushing, grabbing, slapping, throwing objects at) from a caregiver or fear of physical abuse	[PHYSICAL] … I have raised my hand to my kids, but I’m not someone who spanks them for every little thing. No, only if they do something that really gets to me
SEXUAL: Child experienced sexual abuse	No examples of sexual abuse
EMOTIONAL: Caregiver insulted, humiliated or put child down	[EMOTIONAL] What do I admire about my son? Well, his serenity, because he is always calm even when he’s reprimanded. Because he's not perfect, you know. So even when my husband needs to reprehend him he remains mellow. He just listens quietly, he doesn’t try to talk back, because you know how some kids will be argumentative. And I do tell him to defend himself if his father is scolding him for something he didn’t do, but he’ll reply, “Well, I don’t know. He may get just angrier.”
Original Adverse Childhood Experiences: Neglect	
EMOTIONAL: Child felt unsupported, unloved or unprotected PHYSICAL: child lacked appropriate care by a caregiver	No specific examples of emotional or physical neglect [NEGLECT]: For me, it’s difficult. When you work in the fields, it’s hard because you have to take care of your kids. And that’s why it’s hard to get them up early, take them to child care and pick them up in the afternoon. So, sometimes you do neglect them a little bit, because of that Interviewer: Can you tell me more about what you mean when you say “neglect”? Mother: Because you can’t take them to school or pick them up, and that’s when sometimes you feel like you’re not paying enough attention to them. That’s where I feel like I sometimes kind of neglect her
Original Adverse Childhood Experiences: Household dysfunction	
INCARCERATION: Child lived with a Caregiver who went to Jail	[INCARCERATION] His dad went to jail and it was difficult for me to support 4 kids…
MENTAL HEALTH: Child lived with caregiver with mental health issues	[MENTAL HEALTH] We talk little about [emotional health] because I don’t feel he suffers much from that. But I say, I also don’t want to talk much about it because my husband suffers from stress issues and anxiety…. So I do address it, how are they doing, etc. but not that much
SUBSTANCE USE: Child’s caregiver with or ever had problem with too much alcohol, street drugs or prescription medications use	[SUBSTANCE] Right now, his dad is in rehab. He was using drugs. He’s not in touch with them…It has affected them because of our financial situation. Sometimes they see that I’m more stressed sometimes, like when I had a lot of bills to pay
INTIMATE PARTNER VIOLENCE: Child has seen or heard caregiver being screamed at, sworn at, insulted, humiliated, slapped, kicked, punched, beaten up or hurt with a weapon by another adult	[INTIMATE PARTNER VIOLENCE]…Sometimes his dad uses hurtful words with me, and [my kids] don’t like that. It hurts them. And they tell their dad: “Please don’t say that to mom.” And my husband, I believe perhaps he didn’t receive the attention he needed. And he doesn’t do bad things, but he says things that are not right
RELATIONSHIP: Child’s caregivers ever had a change in relationship status (separated, divorced)	[RELATIONSHIP] Interviewer: What is her greatest challenge? Mother: Well, her dad leaving us. I feel this really affected her, because she was already 9 years old. So she was the one who was most aware of what was going on and she felt, well, I didn’t comment about it until she turned 15. No…when she turned 14 and then she asked me what happened. "Well, he left and I didn’t say a word. But I was very sad."
Expanded Adverse Childhood Experiences	
COMMUNITY VIOLENCE: Child seen, heard, victim of violence in neighborhood, community, or school?	[COMMUNITY VIOLENCE] What can I tell you? The thing is, right now there's a lot of.danger in the streets… Because we’re always hearing that there are shoot-outs somewhere. Sometimes I drop my other child off at school and we see patrol cars circling the streets, houses, and all that
DISCRIMINATION: child experienced discrimination?	[BULLYING/DISCRIMINATION]… There was bullying problem, that was when [my daughter] regressed a lot [se me hizo bien chiquita]. Her self-esteem went down a lot, and it was a little difficult to cheer her up, and all that. But I know she can do it. She can do it
HOUSING: child every had problems with housing?	[HOUSING INSECURITY] I researched apartments and I applied for a lot of them, but … we weren’t accepted because of our credit history… so I thought, if we don’t find anything else, we’ll either go to the homeless shelter or we’ll live in our car until we can find a place that’s appropriate for my daughters. Because I was thinking about their school, and sometimes I have to leave them at home by themselves, so I want them to be somewhere safe. And someplace where they won’t be exposed to abuse, somewhere I can feel comfortable leaving them
FOOD: Child did not have enough food to eat or food ran out before could buy more?	[FOOD INSECURITY]: "Kids, we’re going to go to the store, but there isn't enough money."
SEPARATION: child separated from caregiver due to foster/immigration?	[SEPARATION] When I came here, I was alone at first. I came here alone at 19, and I left my small children behind. I came here seeking a better life, like many people. I came here to work, and I was alone for six years, working… but since things were so complicated in Mexico, I thought, “If I could make it on my own there, I don’t see why I couldn’t make it there with my kids.” So, I thought that if I came back here I would bring them with me
DISABILITY: child lived with caregiver with serious physical illness or disability?	[DISABILITY] …She didn’t want to face her dad. She said that he wasn’t her dad anymore, that she wanted a dad who would take her to school, who would spend time with her. But since her dad could no longer do those things, she said he wasn’t her dad anymore, that she wanted a different dad, a good dad, because she didn’t want to have a bad dad anymore. And I would tell her, “It may be good or bad, but he can’t take you to school or pick you up anymore because he’s sick.” But sometimes my husband would say, “Take me in the wheelchair.” So, I would take him with the wheelchair, and she was so happy when she saw him. She would say, “You made it! Tell mom to push you so you can come."
DEATH: child lived with caregiver who died?	[DEATH] Interviewer: What do you think are your daughters greatest challenges/difficulties she faces? Mother: The loss of her father. That was what’s affected her the most. Very much so. Because when my husband died, he was murdered; so he wasn’t sick, he was alright. So then this happens all of the sudden…and that’s something very sad for an 11-year-old kid… Emotionally speaking this is something that affects them very much

### ADVERSITIES

#### Expanded ACEs

Mothers described the community and structural adversities identified in the Expanded ACEs – (housing instability, food insecurity, witnessed violence) more commonly than they described events from the original ACEs (Table 2). Mothers worried most about community safety with fear of shootings, bullying, and gang violence.

One mother shared,

“There was a time when...a car would pass by and they would shoot people at random. … I moved away from that area for that very reason, mostly for my kids, because I didn’t want them to see all that.” (37-year-old, mother of 4, living in the U.S. for 18 years)

In addition to concerns about physical injuries to their children from community violence, participants also worried that their children would be mislabeled as perpetrators of violence. For example, mothers shared stories of their children’s siblings (who were not study participants) being collateral damage in drive-by shootings or mistakenly detained by law enforcement. While these adverse events were not described as happening directly to the youth in *A Crecer*, they had marked impacts on the entire family.

Another frequently cited Expanded ACE was housing insecurity and families sharing housing with other families to afford rent. A mother shared, “As I said, we used to live in a house that we lost. We were renting, but the rent went up … I had to find something we could afford, so we moved to the trailer and that’s where we are now." (46-year-old, mother of 4, living in the U.S. for 25 years shared).

#### Immigrant experiences: structural racism

Mothers also highlighted structural challenges, including ways that they tried to access resources to mitigate structural adversities. For example, mothers described the systemic adversity their children experienced in school, including differential treatment by teachers, and the effects that systemic racism had on resources allocation and availability. A mother explained,

“[That school] was more American than Mexican... they used to say it was the best school, because only Americans went there. now there aren’t as many Americans going there, *because* there are more Mexicans. And maybe the Americans have left. Well, sometimes Americans are racist. [They left] so that they can be with their same… people from the same country as them. Students as well as teachers. [But] you also learn from the Americans... You get a better education.” (46-year-old, mother of 4, living in the U.S. for 25 years)

Mothers shared that even beyond primary schooling, the sociopolitical climate led to feelings of instability and limitation for immigrants. Mothers expressed concern that their child’s immigration status would limit their access to resources, programs, and education. One mother shared,

“Some presidents are stricter, they change the rules, and all that keeps a lot of people stuck…[my daughter] starts thinking about those things, it’s like she feels like her hands are tied. But I tell her that everything might be better in the future... Just like they said, 'Take away DACA,' they can restore it. They can say 'all of those papers that are at a standstill,' like ours, they can open up them and get things moving overnight. She should fight for what she wants, and fate will decide what happens in the future to clear a path for her.” (36-year-old, mother of 4, living in the U.S. for 14 years)

#### Immigrant experience: Monolingual Spanish

Mothers shared limitations in accessing resources as monolingual Spanish-speakers. Some mothers described limitations in helping children with schoolwork, accessing structural resources, or even limiting their ability to become United States citizens. A mother stated that she would like to learn to speak English, “to… be more informed and not have to wait for [my children] to come home when a document comes in the mail; I’ll be able to read it by myself. And then I’ll be able to say, ‘No, I can fill out this application myself.’” (46-year-old, mother of 4, living in the U.S. for 25 years) Participants cited barriers in communicating with their teens and limitations in their abilities to understand their children’s conversations with friends and siblings. A mother also saw her child struggle in school as an English-as-Second-Language student, “He didn’t speak [English] either when he first started school. And yes, that is a challenge, perhaps even to this day. And all the classes they take, they can perhaps be challenging because they don’t speak the language. So that’s a difficulty for them, the language.” (38-year-old, mother of 3, living in the U.S. for 17 years).

#### Original ACEs

Beyond structural challenges, mothers identified adverse experiences in their families that were included in the original ACEs, as well as other experiences such as intergenerational trauma and effects of immigration on the family, which were not among the original ACEs. Mothers did not report caregiver abuse or neglect of their children, a marker in the original ACEs. Within the original ACEs framework, mothers shared a few cases of incarceration/detainment of fathers and rare cases of interpersonal violence or substance/alcohol use by biological fathers (Table 1). One mother described how she framed paternal incarceration for her son:

“My husband got out of jail… he asked to see [my son] ...I took [my son] there so he would learn, so that he would see that everything in life has consequences, what you do. And that’s why I said, ‘You need to think three times before you act.’ And that also helped [my son] a lot..” (38-year-old, mother of 3, living in the U.S. for 19 years)

#### Intergenerational trauma

Many mothers also discussed intergenerational trauma including their own adverse childhood experiences. These stories often highlighted mothers’ abilities to overcome and achieve despite their challenges. A mother shared: "My mother was also a single parent. She separated from my dad because he beat her pretty badly…and despite all that, my grades were not bad.” (48-year-old, mother of 5, living in the U.S. for 20 years).

#### Immigrant experiences: separation from resources

Mothers described multiple impacts of immigration on the family including parent–child separation, depletion of finances and changes in familial support systems. Parent child separation occurred in several ways. One example includes the inability for families to migrate together leading to more permanent separation. A mother shared, “[My son] only knows his dad by pictures. But his dad can’t come here. And my son can’t go there either. These are things that have affected him.*”* (48-year-old, mother of 5, living in the U.S. for 20 years) There were several examples of mothers immigrating ahead of their children to save money, staggering migration to reduce costs in the United States. A mother stated,

“It was difficult for me to support four kids… I came here every year to work in the lettuce industry, and then I would go back… I left [my son] there, when he was three months old, in Mexico. He was a newborn. I left him there for six months when I came to the U.S., and then I brought him here when he was nine months old. That was the only time I was far from him, when he was a baby.” (48-year-old, mother of 5, living in the U.S. for 20 years)

Some families separated due to seasonal migrant farm work while others declined migrant work and accepted a lower standard of living in order to remain together. Once mothers decided to bring their children to the U.S., those children left behind their support systems which might have included another parent, siblings, grandparents, and extended family members who may otherwise have been able to provide emotional support and help buffer against financial hardships. A mother shared the most important people in her child’s life: “[My daughter] always said it’s her siblings and me. It’s the three of us. And also, her grandfather, who lived for a short while with us. And I do see her staying in touch with him…they talk often. And he accompanied her from the time she was 7 until she was 10…and then the man had to go back to Mexico. But she continues to care for him very much.” (37-year-old, mother of 3, living in the U.S. for 17 years).

This loss of family support systems left some mothers struggling with the need to work long hours while wanting to spend quality time with their children. A mother explained, “We need to spend more time with them, talk to them. So, they can share their thoughts with us. In my case, I would rather spend more time with my kid than at work. But honestly, I worked over ten hours, then came home, went to [my daughter’s] school. So, after all that, I wasn’t able to spend any time with [my son].” (48-year-old, mother of 5, living in the U.S. for 20 years) Additionally, the impact of losing family support systems seemed to “mature” children, according to a mother, “[My son] started to like, let go of his childhood. Because when I brought them here, they had been with [his grandmother], and people they knew. And of course, when you move them somewhere else, kids go through changes: their behavior, the way they are." (38-year-old, mother of 3, living in the U.S. for 19 years).

### Protections

#### Immigrant experiences: “Pull Factors”

Mothers described immigration as a necessity and opportunity for growth for their families. Mothers shared factors that pushed them towards immigration to the U.S., including poverty, limitations in economic advancement, and safety concerns. They shared “pull factors” including a desire for opportunities for their children, safety, and breaking away from cultural constraints.

A mother emphasized, “That’s what I tell my daughter. ‘I want to see you be successful in this country, because we come from a very poor country, Mexico. And we also suffered a lot during our childhood. I don’t want that for you.’ I want my daughter to be successful… in the career she chooses.” (46-year-old, mother of 4, living in the U.S. for 25 years).

Mothers saw the chance for children to break out of cycles of poverty. A mother shared:

“...being here enables us to offer our children a better life… Here we can buy things that we weren’t able to back home because here there are jobs. … Raising a family here in Salinas makes it possible for them to have healthcare, better schools, and we’re better able to take care of their needs.” (53-year-old, mother of 2, living in the U.S. for 24 years)

#### Bridging resources

In navigating the challenges that arose for their families, mothers worked to find protective community programming for their children by engaging with schools and mental health care.

A mother shared how she accessed support for her daughter through health care:

“Her dad leaving us - I feel this really affected her, because she was already 9 years old… I went to the doctor and told the doctor that I wanted [my daughter] to be referred to a psychologist so she could talk and let all those feelings out… And so, we did, we did some therapy and I feel it was very useful, so she could release all the feelings she had” (37-year-old, mother of 3, living in the U.S. for 17 years)

#### Parenting strategies

As illustrated, participants in this study navigated the challenges and strains in their lives as mothers, often thriving, despite experiencing a wide array of personal and structural challenges. As a result of the original and Expanded ACEs that these mothers overcame, many of them reported working to promote resilience in their children through specific lessons and skills including: teaching life-lesson, demonstrating their own resilience, accessing spirituality, and drawing on familismo.

##### Teaching life lessons

Mothers explicitly taught children to move forward through difficulties and access outside support. A mother said,”I tell him, ‘Don’t look back.’ …You have to make your own way, because there is a lot of help here, a lot of information. There’s no reason for us to be left behind; we need to keep going forward.” (33-year-old mother of 3, living in the U.S. for 15 years).

Mothers communicated the need to cultivate future-orientation and hard work to achieve goals. One mother shared,

“I really like [Salinas] because of the farmland, the work, seeing how crops are planted and harvested, I use that as an example of how preparing the land to grow a crop is the same thing we do as parents with our children. I like the kids to see what the fields look like before they’re planted and how they look after they’ve been planted. I always make the comparison, 'Look, when the soil isn’t fertile, it’s sad and dull. It doesn’t look good. A person is the same way: if you don’t study you won’t produce; you’re a person without meaning.’” (42-year-old, mother of 3, living in the U.S. for 18 years)

Another mother highlighted the importance of perspective building to focus on long-term success, “I always tell him…’you will succeed, I know you’ll make it and later on you’ll look back and this struggle will seem very distant.’” (38-year-old, mother of 3, living in the U.S. for 17 years).

Mothers discussed how they led by example to foster resilience and self-care. A mother explained that she tells her children, “Well, what I do personally when I’m really feeling stressed is go for a walk. I clear my mind and think about things, and when I get back, I can start doing whatever I need to do feeling calmer.” (46-year-old, mother of 4, living in the U.S. for 25 years).

Some mothers discussed sources of stress they have experienced and suggested coping mechanisms to overcome challenges. Mothers also used their own challenging life experiences to motivate children, “I took both of my daughters [to work in the fields]. She says … ‘I don't want to go back Mom; it's really hard and very poorly paid. There are jobs that pay more and require working less.’ …It serves them as an experience.” (36-year-old, mother of 4, living in the U.S. for 14 years).

##### Spirituality as a tool for resilience

Mothers also explained how they drew on their own spirituality to teach their children to maintain perspective that even in times of limitation, sharing and caring for community is important. A mother reflected,

“I’m inside a trailer and I’m cold. Do [unhoused people] not feel cold? It’s wet, and they have nowhere to get cleaned up and get a hot meal. I thought about that a lot, and several times I even made food and coffee and took it to them. I thought, “I don’t have much to give, but I thank God for giving me a roof over my head and food for my children.” (46-year-old, mother of 4, living in the U.S. for 25 years)

Mothers also felt spirituality was an important source of strength and community for themselves and families. A mother shared “And I thank God that when I was in pain and anguish, God was my friend, my strength, my rock.” (46-year-old, mother of 4, living in the U.S. for 25 years).

##### Familismo

Almost every mother cited family as a critically important source of support for a child. Mothers stated that a strong foundation starts with parents who support each other and set examples for their children. A mother shared examples of family working as a collective. Mothers shared illustrations of this, including young adult children supporting families financially or emotionally supporting younger siblings. Mothers reported that quality time as a family was an important buffer against bad influences. A mother shared, “In order to be happy, there needs to be a lot of communication, and particularly respect and affection in the family environment, especially with the parents. And to just spend time with them, make time for them, which is what kids need most: some quality time.” (42-year-old, mother of 3, living in the U.S. for 18 years) However, for some parents, this became difficult when raising a child in the U.S. in a more individualistic culture that felt in opposition to familismo norms. A mother noted, “they want to go places with their friends, ‘Give me permission,’ and go out for a walk and things like that. But not me. In my culture, you couldn’t do that until you were a certain age, and you knew how to take care of yourself, then you were allowed.” (42-year-old, mother of 3, living in the U.S. for 18 years).

#### Intergenerational resilience and breaking cycles of violence

Having experienced intergenerational trauma and systemic oppression, mothers discussed their strategies for improving educational and socioeconomic opportunities for their families. A mother shared,

“My mother is from a small town… the highest goal that she taught me to have for myself was to marry and have children. And well, unfortunately that's what I did.... So, the truth is I didn't want [my daughters] to be like me. I wanted them to grow a little more. So, we struggled, and we came here. But that was my motivation. I didn’t want them to get stuck there or for their goal be to marry and have children.” (36-year-old, mother of 4, living in the U.S. for 14 years)

Mothers spoke overwhelmingly about breaking cycles of violence in the home and discouraging violence at school. They highlighted the importance of communication as the most important aspect of guiding a child’s development. Some described experiencing authoritarian parenting during their own childhoods and wanting to parent with an emphasis on clear, respectful communication. A mother shared “Unfortunately, parents often talk to their kids in a violent way…and they absorb that violence. But we need to council them, not scold them, but provide advice to them.” (37-year-old, mother of 4, living in the U.S. for 18 years).

## Discussion

This study contributes the needed literature on the adverse events experienced by immigrant Latinx mothers and their children including the impacts of structural racism, political instability, intergenerational trauma, depletion of financial resources, loss of family support systems, and family-child separation related to immigration and migration for work. Mothers detailed their families’ strategies for resilience-building and protection of their children. The Adverse Childhood Experiences that these mothers identified, are likely to impact life-course trajectories and health related outcomes of children in immigrant communities and should be considered in the measurement of ACEs. Many of these more immigrant-specific ACEs result from policy and structural factors that could be changed to promote child health. In addition, interventions to build resilience and buffer the impacts of all ACEs can be informed by the collective wisdom of the parents and families who work to protect their children from adverse events.

This study shows the need to consider immigrant-specific ACEs that are not captured or adequately delineated in current ACEs measures. The findings of this study are consistent with prior research showing that in socially disadvantaged populations, Expanded ACEs are more pervasive than the original ACEs[3]. However, our findings suggest that single measures on the current ACEs framework are capturing multiple immigrant-specific experiences making risk categorization for low, intermediate, or high risk for toxic stress inaccurate. For example, child separation from a parent in an immigrant family goes beyond child protective services and foster homes, as measured by existing ACEs assessments, and instead may be capturing multiple events including separation due to staggered migration, seasonal migration, deportation, or ICE detainment. Current ACEs measures also fail to capture separation from family members such as siblings and extended family members, critical to Latinx family structures. Additionally, the unstable political climate leading to family separation, and lack of health care contributes to significant impacts on available resources and increasing number of ACEs in families. Studies of quantitative measures of immigrant-related ACEs currently focus on youth who have immigrated themselves [18], but the mothers in this study highlight ways in which immigrant-related disparities persist even among U.S.-born children living in immigrant communities. The current framework for ACEs does not capture the full dimension of systemic and immigration-related adversities.

As suggested by our findings, mothers found ways of helping their children navigate these barriers and worked to protect them against adverse experiences through individual, family, intergenerational and structural means. Similar to prior research, mothers leveraged interpersonal resilience by exhibiting their own resilience and by teaching life lessons. Prior research has shown that immigrant women with strong self-esteem, self-mastery and personal agency had strong desire for employment, education, and autonomy [40]. Similar to the mothers in our study, other research underscores ways mothers also harness the protective effects of familismo and extend family support to create positive ethnic identities [41]. Familismo has been associated with promotion of the social and economic stability of the entire family system [42]. Importantly, mothers in our study built upon intergenerational resilience by breaking cycles of violence and focusing on their excellent communicative parenting. A study of Latinx parents in North Carolina reported parent–child communication is a tool used by immigrant parents to help promote resilience in the process of adaptation [43].

The adverse events identified by the mothers in our study, including housing insecurity, neighborhood safety & violence, school bullying, and separation from a parent due to immigration, are socially-modifiable experiences that can be alleviated or buffered through policy driven changes [44]. Recently, several states have advanced policy to address these gaps by increasing minimum wage and employment opportunities, expanding community and domestic violence prevention initiatives, and working to keep schools open and accessible during the COVID19 pandemic [45]. In an effort to reduce community violence in communities of color, Boston, New York City, and Chicago have demonstrated that summer youth employment programs have longer term impact on reduce crime and incarceration in these communities of color [46–48]. In 2019, California expanded access to healthcare for undocumented children up to the age of 26 which improved access to care and reduced financial strain [49]. Further, in 2022 California’s governor proposed expanding Medi-Cal access to all undocumented Californians who are financially eligible, opening access to health and mental health care for parents in these families as well [50]. Related to this, in a study assessing pre- and post- DACA effects on healthcare access from 2009 to 2016, findings showed improved insurance coverage and fewer delays in care because of financial restrictions [51].Future work is needed to examine whether immigrant ACEs correlate with poor health outcomes similarly and in a dose-dependent manner as those assessed in the current ACEs framework. Research is also needed to evaluate if any impact of these ACEs persist beyond first- and second-generation immigrant children. This qualitative study could inform further development of quantitative measures of immigrant ACEs that could be assessed in a larger study to examine these relationships. Although state-level policies have mitigated some of the immigrant ACEs we identified, Latinx youth remain at risk in the absence of national policy designed to mitigate systemic adversities experienced by this population.

While our study adds to the literature supporting a need for additional study of immigrant-specific ACEs and interpersonal protective factors, our study has several limitations. Although data saturation was achieved, the sample in this study is small, limiting its generalizability. However, our sample’s ethnic profile is similar to other established migration destinations for agricultural work in California and other regions making the sample relevant to the rapidly growing populations of Latinx in rural communities around the country [52]. Second, mothers were not asked specific measurable adverse childhood experiences, but rather through their experiences immigrating and raising families, these experiences were elicited. Participants lived in a relatively immigrant-friendly California community with expanded social services for undocumented immigrants which may reduce the adverse experiences they have compared with immigrants in other states or communities. Fourthly, some participants may be hesitant to discuss sensitive topics or disclose controversial or differing viewpoints. Despite these limitations, the depth of protective factors and immigrant related adverse experiences is noteworthy and requires additional public health attention.

## Conclusion

This study elucidates immigrant-related experiences that contribute to inequities known to be important to adolescent health and wellbeing. Interviews with Latinx mothers of adolescents, underscore key immigrant-specific adversities that should be considered in extending the ACEs framework to account for the full health impacts of ACEs in immigrant communities. Screening for additional immigrant-specific ACEs could allow health providers to identify young people at risk for developing an array of poor health outcomes. Clinicians and researchers can partner with families in immigrant communities to amplify their efforts to build resilience in their children and buffer the impacts of all ACEs by building identity, drawing on family supports, leveraging communication, accessing education, and addressing intergenerational trauma. These approaches offer insightful direction for future community-tailored interventions can build on this foundation to reduce health disparities and promote health equity.

## Data Availability

The datasets generated and analyzed during the current study are available from the corresponding author on reasonable request.

## Abbreviations

ACEs: Adverse Childhood Experiences
